# Resistance training partially restores age-related differences in skeletal muscle amino acid transporters - secondary analysis from two randomized controlled trials

**DOI:** 10.1016/j.jnha.2026.100808

**Published:** 2026-02-21

**Authors:** E. Lander, H. Hamarsland, M.J. Lees, D. Moore, T. Raastad

**Affiliations:** a Department of Physical Performance, Norwegian School of Sport Sciences, Oslo, Norway; b Inland Norway University of Applied Sciences, Lillehammer, Norway; c Faculty of Kinesiology and Physical Education, University of Toronto, Toronto, Canada

**Keywords:** Aging, Anabolic resistance, Amino acid transporters, Resistance training, Muscle biopsy

## Abstract

**Objective:**

Postprandial stimulation of muscle protein synthesis depends on intracellular amino acid (AA) availability and effective transmembrane AA transporters (AATs). AA transport may be impaired in sedentary and older adults. We compared skeletal muscle AATs between young and older adults and examined effects of resistance training combined with increased protein intake.

**Design:**

Secondary analysis from two randomized controlled trials

**Setting:**

Participants were enrolled in trials comparing milk and native whey effects on anabolic signaling, muscle mass, and strength.

**Participants:**

Healthy young (n = 32; 14♀/18♂, 20–45 yrs) and older (n = 28; ♀/17♂, 70–80 yrs) adults

**Intervention:**

Whole-body progressive resistance training 3×/week for 11–12 weeks with protein supplementation.

**Measurements:**

Pre- and post-intervention assessments included lean leg mass (LLM), one-repetition maximum (1RM) leg press, and AAT protein levels in *m. vastus lateralis* biopsies. Western blots quantified L-Type Amino Acid Transporter 1 (LAT1) and 3 (LAT3), 4 F2 heavy chain (CD98) and solute carrier 38 member 9 (SNAT9) in cytosol (_C_), membrane (_M_) and nuclear (_N_) fractions. LAT1 membrane (IF-M) and intracellular (IF-IC) distribution were assessed by immunofluorescence.

**Results:**

Training increased LLM by ∼1 kg and 1RM leg press by ∼31% in both groups (p < 0.001). At baseline, older adults showed higher SNAT9_M_ and IF-M LAT1 and lower LAT1_C_ versus young (p < 0.05). Training produced age-dependent changes: LAT3_C_ increased in young (p = 0.39) and CD98_M_ increased in old (p = 0.26) yielding significant time × age interactions (p < 0.05). Across groups, training reduced LAT1 intensity and SNAT9_M_ and increased CD98_N_ (p < 0.01−05). In young participants, IF-IC LAT1 decreased 9 ± 14% (p < 0.05) and CD98_N_ increased 59 ± 97%(p < 0.01). Posttraining, older adults displayed higher IF-M LAT1 and lower CD98_M_ than young (p < 0.05).

**Conclusion:**

Resistance training with protein supplementation improved muscle mass and strength and modified AAT profiles. Age was associated with higher membrane LAT1 and SNAT9, while training attenuated some age-related differences and produced distinct effects on LAT3 and CD98 by age. Exercise may partially counteract age-related alterations in muscle AA transport, with implications for muscle health in aging.

## 1 Introduction

Ingestion of high-quality protein stimulates the uptake of amino acids, anabolic signaling, and ultimately protein synthesis in healthy skeletal muscle [1], which is enhanced in combination with exercise [[2], [3], [4]]. Transmembrane amino acid transporters (AAT) facilitate the transport of amino acids from the circulation into the muscle cell and its compartments [5,6], making them vital for supporting muscle protein synthesis. Lower capacity or malfunction in AATs could reduce amino acid availability, leading to attenuated muscle protein synthetic rate in response to feeding, termed anabolic resistance, which is reported in relation to aging, inactivity and cancer [7]. Although slower uptake of amino acids into skeletal muscle have been reported in older compared to younger adults [[8], [9], [10]], the AATs role as a potential contributor to anabolic resistance has received limited attention in humans [11].

AAT protein levels are, in general, upregulated through the activation of the general amino acid control (GAAC) pathway when energy availability is low, but can also be upregulated by exercise or protein feeding [11,12]. In physically active young and older adults, it has been reported both higher [13] and indifferent [14] levels of L-Type Amino Acid Transporter 1 (LAT1) in the older group. Interestingly, one study has reported higher muscle LAT1 levels in young males compared to females irrespective of training status [15], indicating potential sex differences. LAT1 muscle protein levels have been reported to increase [16] or remain unaltered [15] by resistance training in young adults, but has not been investigated in older adults. By contrast, seven days of bed rest blunted protein feeding-induced increases in muscle LAT1 in older adults [17]. The presence and function of AATs in muscle may therefore be different for active and inactive individuals, and follow-up studies are necessary to confirm whether ageing is a factor affecting the levels and regulation of AATs.

AATs facilitate the intracellular uptake of amino acids when fixed in the membrane. In older men, intake of 12 g whey protein after resistance exercise increased muscle membrane protein levels of LAT1 and sodium-coupled neutral amino acid transporter 2 (SNAT2), with no change in proton-assisted amino acid transporter 1 (PAT1), whereas ingesting 28 g resulted in increased SNAT2 and PAT1 and no change in LAT1 protein levels [18]. Ingesting 0.25 g·(kg body mass)^−1^ crystalline amino acids alone or following acute strength training did not change sarcolemmal LAT1 content in young men [19], suggesting that sufficient protein intake can stabilize acute changes in LAT1 by strength exercise. This could translate into long-term adaptations, as seen with unaltered LAT1 protein in young adults after eight or twelve weeks of strength training combined with whey protein supplements corresponding to 0.3 g·kg^−1^ or 26.3 g protein respectively [15,16]. On the other hand, PAT1 protein increased and SNAT2 protein remained unchanged following 12 weeks of strength training regardless of protein or placebo supplementation [16]. Different AATs may therefore respond differently to strength training and protein intake.

At least 66 AATs are identified in human homologs [11], but potential differences between young and older adults remain sparsely studied. Leucine is a principal stimulator of protein synthesis [20], mainly transported by LAT1 which requires the 4 F2 heavy chain (CD98) for activity [21]. The L-Type Amino Acid Transporter 2 (LAT2) transport leucine similarly to LAT1 but exerts greater importance for glutamine efflux and in equilibrating the relative amino acids concentrations [[22], [23], [24]]. The L-Type Amino Acid Transporter 3 (LAT3) is notable for regulating intracellular leucine levels in prostate cancer cells and may mediate branch-chained amino acid efflux [25,26]. The lysosomal solute carrier 38 member 9 (SNAT9) can modulate the Mechanistic Target of Rapamycin Complex 1 (mTORC1) activity and, unlike PAT1, transports leucine [27,28]. Investigation of LAT3 and SNAT9, alongside LAT1/CD98, could sharpen our understanding of postprandial muscle amino acid, and leucine particularly, availability.

Blunted muscle anabolic response to protein intake may stem from limited substrate availability caused by dysfunctional, or reduced, AATs. We therefore hypothesized that older adults have lower abundance or altered distributed AATs versus young adults. Because resistance exercise increases utilization of dietary amino acids for protein synthesis [29] and supports hypertrophy [30], we hypothesized that long-term strength training mitigates age-related AATs differences. We therefore assessed AAT protein abundance and subcellular localization and adaptation to strength training in young and old participants.

## 2 Material and methods

### 2.1 Participants and ethical approval

Twenty-eight old (70–80 yrs) and thirty-two young (20–40 yrs), healthy and untrained men and women were included in this study (Table 1). All participants were a part of a more extensive study investigating the effect of milk and native whey and whether short-term differences in anabolic signaling and muscle protein synthesis would translate into long-term muscle mass and strength adaptations. The study was evaluated and approved by the Norwegian School of Sport Sciences (NSSS) Ethical Committee (ref 2012/284), as well as the Norwegian Center for Research Data. A short summary of the design and protocol will follow, and complete information about the design and protocol can be found in the referred articles [31,32]

**Table 1:** Subject characteristics for the groups are presented in mean ± SD, and p-values for comparisons of baseline values between cohorts.

	Young cohort	Older cohort	p-value
Participants (N)	32 (F = 14, M = 18)	28 (F = 11, M = 17)	
Age (years)	29.1 ± 5.6	73.8 ± 2.9	<0.01
BMI (kg/m^2^)	24.8 ± 3.6	25.0 ± 4.0	0.94
Body Mass (kg)	79.4 ± 13.2	75.5 ± 15.2	0.29
Fat mass (kg)	22.7 ± 8.0	23.0 ± 8.2	0.86
Lean body mass (kg)	54.0 ± 8.9	49.9 ± 9.8	0.09
Lean leg mass (kg)	19.1 ± 3.4	17.3 ± 4.2	0.06
1RM Leg press (kg)	270 ± 75	165 ± 47	<0.01

N = number of participants. F = female. M = Male.

### 2.2 Study design and protocol

This mixed factorial, non-randomized, study compares a young and old cohort, from participants taking part in a double-blind, randomized, controlled trial. The participants received two daily servings of either milk protein manufactured by Tine ASA (Oslo, Norway) or native whey purchased from Lactalis® (Laval, Mayenne, France) containing 20 g of protein, during a 11- or 12-week traditional whole body strength training intervention, for the old and young respectively [31,32].

### 2.3 Muscle biopsies and preanalytical processing

Resting muscle biopsies were collected 1 h after eating a standardized breakfast (followed by an overnight fast) and with no strenuous physical activity the last 3–4 days, from the mid-portion of *m. vastus lateralis* before and after the training intervention for all participants. The procedure was performed as previously described [33]

Approximately 50 mg of muscle tissue was fractionated to the cytosol (_C_), membrane (_M_) and nuclear (_N_) fractions, using a commercial fractionation kit according to the manufacturer's procedures (ProteoExtract Subcellular Proteo Extraction Kit, no. 539790, Calbiochem; EMD Biosciences GmbH, Schwalbach, Germany). Subcellular fractions have been validated in our lab with western blot on several occasions to confirm successful fractions with glyceraldehyde-3-phosphate dehydrogenase (GAPDH) for the cytosolic fraction [34], poly ADP-ribose polymerase 1 for the nuclear fraction and cyclooxygenase-2 for the membrane fraction. Protein concentrations were assessed as previously described [33], with an acceptation of the coefficient of variation (CV)<10%.

### 2.4 Immunohistochemistry

Serial cross-sections (8 μm) were prepared from OCT-embedded samples onto room temperature uncoated glass slides and left to air dry for 10 min to remove excess crystallized water. Sections were fixed at −20 °C and then washed for 3 × 5 min in phosphate buffered saline (PBS, Sigma-Aldrich, D1408) supplemented with 0.2% Tween (PBS-T, Bioshop, TWN510.500) to remove fixation reagent. An A-PAP pen (Sigma-Aldrich, Z672548) was used to draw a hydrophobic barrier around the prepared sections. Sections were then blocked before incubation in primary antibody for LAT1 and Myosin Heavy Chain I (MHCI) for two hours at room temperature. A summary of the antibodies used, and concomitant protocols is provided in the Supplementary Materials (Supplementary Table S1).

Following incubation, sections were washed for 3 × 5 min in PBS-T and incubated in the appropriate secondary antibody for 1 h at room temperature. The sections were then washed 3 × 5 min in PBS-T and incubated with Wheat Germ Agglutinin (WGA-350) for 30 min at room temperature to mark the sarcolemmal membrane. After a final wash (1 × 5 min) in PBS, slides were left to air dry for about 1−2 min at room temperature until the visual water stains evaporated.

Sections were mounted with 20 μL DAKO fluorescent mounting medium and sealed by glass coverslips to protect the muscle sections and to preserve fluorescence signals. Slides were left overnight in a dark room before observation.

### 2.5 Image capture and processing

Image capture was performed using an EVOS FL Auto Cell imaging microscope (Thermo Fisher, Waltham, MA) at × 0.40/75 NA magnification, using the automated image capture and stitching functions, as previously described [19]. Slides were prepared with all sections from a single participant in series and all imaging parameters were kept constant (i.e., exposure time, gain, light intensity). For each participant ∼100 fibers from each time point were counted and were available for subsequent analysis.

**Fig. 1:**
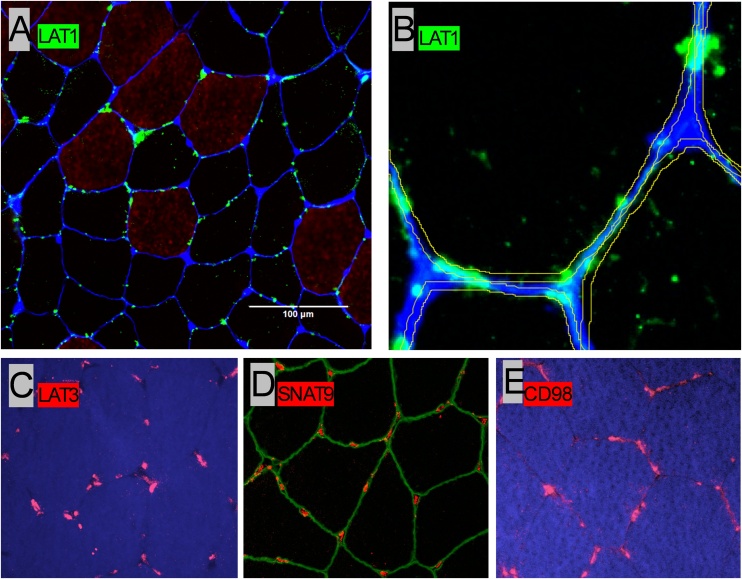
Representative image of immunohistochemistry analyses. On top left side is the combined image of L-Type amino Acid Transporter 1 (LAT1) in green color, Myosin Heavy Chain 1 (MHC1) in red color and sarcolemma membrane in blue color. On top right side is a visualization of the peripheral localization of LAT1 distribution defined as membrane (A and B). On the bottom is the visualization of the AATs LAT3 (C), SNAT9 (D) and CD98 (E) in red color, which were analyzed with western blot only, shown in Fig. 4 (immunofluorescence method not described).

LAT1 visualization was conducted as described previously [35] with WGA and MHCI demarking the cell membrane and Type I fibers, respectively (Figure 1). The peripheral localization of LAT1 (membrane region) was quantified as the LAT1 signal intensity within the outer 1.5 μm of each fiber, whereas the intracellular (IC) localization of LAT1 was quantified as the LAT1 signal intensity in the remainder of each fiber. Membrane-to-intracellular ratio (M/IC-ratio) was calculated as ‘membrane’ LAT1 signal intensity expressed in relation to LAT1 signal intensity in the remainder of each fiber. All immunofluorescent image analysis was conducted on ImageJ software (Fiji plugin, v. 1.5, National Institutes of Health, Bethesda, MD, USA).

### 2.6 Western blot

Gel electrophoresis and western blot were performed to investigate the total protein content. The samples were diluted with ultra-pure water (dH2O), Laemmli Sample Buffer (4×, Cat. #161-0747, BioRad, CA, USA), and Dithiothreitol (Cat. #161-0611, BioRad, CA, USA) to contain equal amounts of protein per well which were determined by the measured dynamic linear range for each protein of interest in the specific fraction. 5 μl weight marker (Precision Plus Protein All Blue Standard, #1620373, BioRad, CA, USA) was pipetted into the first and last well of each gel.

The fractionated samples were run in singles, including one young and one old participant in the same gel, with duplicated control samples, and with duplicated gels. The loading control sample was made from a mixture of young and old muscle tissue from all biopsy time points. The protocol for each protein investigated, CD98, SNAT9, LAT1, LAT3, Activating transcription factor 4 (ATF4) and eIF2α, is listed in Supplementary Table S2, and identical steps for all proteins are listed below.

The electrophoresis was run with Tris/Glycine/SDS buffer (10x, #1610772) at 200 V for 35 min (Power Pac 200, Bio-Rad, USA) and activated for tryptophan using UV-light for 2.5 min in ChemiDoc ™ MP Imaging System (Bio-Rad, CA, USA). PVDF membranes (cat # 162-0177, Bio-Rad, CA, USA) were activated in methanol for 30 seconds (EMD Millipore Corporation, Billerica, MA, USA), then washed 1 × 30 seconds and 1 × 2 min in dH2O before equilibration in transfer buffer for 15 min. Stacks were equilibrated in transfer buffer for 10 min. Proteins were transferred from gel to membrane in Trans-Blot® Turbo™ Transfer System (cat no. #1704150, Bio-Rad, CA, USA) using Trans-Blot Turbo RTA Mini kit (0.2 μm PVDF Transfer Kit, cat.no#1704272, Bio-Rad, CA, USA).

A stain-free blot image of the PVDF membranes was taken and then cut into smaller portions based on the weight of the appropriate proteins, before blocking procedure (see Supplementary Materials for specific blocking solutions). Then the membranes were washed 2 × 2 min in TBS-T (Tris-buffered saline, 10 x TBS, Cat #170-6435, Bio-Rad, CA, USA and 0.1% Tween 20, 437082Q, VWR) and placed in TBS (Tris buffered saline, 10 × TBS, Cat #170-6435, Bio-Rad, CA, USA) before incubation with primary antibodies at 4 °C overnight on a grate board.

The next day, membranes were washed with TBS-T for 15 min and then 3 × 5 min in TBS before and after incubation in the appropriate secondary antibody for one hour at room temperature. Membranes were then incubated for five minutes with Chemiluminescent Substrate SuperSignal WestDura® (Extended Duration Substrate, Thermo Scientific, Cat # 34076, Rockford, IL, USA) before imaging of protein bands with the ChemiDoc ™ MP Imaging System and analyzed with Image Lab ™ Software (Bio- Rad Laboratories, Hercules, CA, USA). All images were adjusted for total protein in corresponding stain-free blot image and further normalized to the mean of control samples for that gel. A CV < 20% was accepted for control samples within each gel. Representative bands are shown in Supplementary Table S3.

### 2.7 Statistical analyses

Sample size calculations were based on changes in lean mass as described previously [31]. All analyses were done in GraphPad Prism 9.2.0. Subject characteristics were compared between cohorts using baseline values in absolute numbers and analyzed using multiple unpaired t-test. The total number of participants used in each analysis is listed in supplementary Table S4.

To analyze the effect of the training intervention, a two-way ANOVA with repeated measures (time x age) was used on changes in lean leg mass, 1RM and protein levels and intensity. Unpaired t-test was performed to compare the relative changes (%) from baseline to after the training intervention between groups and paired t-test to compare the relative changes (%) in protein intensity between muscle fiber types in the groups separately. Due to smaller sample size when comparing sex differences across age, t-tests were used for analyzes on baseline and post-training values, as well as for the relative change between the two time points.

Two-way ANOVA was further used to compare the acute response in protein levels for young and old separately. Arbitrary units were used when analyzing the acute changes within groups (time x training status), whereas the %-change was used when analyzing differences between cohorts (training status × age). Whenever ANOVA testing showed significant differences (p < 0.05), a post hoc test (multiple comparison tests corrected with Bonferroni with 95% confidence level) was performed.

## 3 Results

### 3.1 Lean leg mass and 1RM

**Fig. 2:**
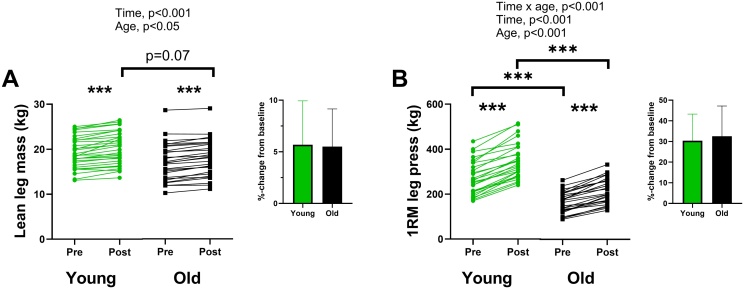
Lean leg mass (A) and 1RM leg press (B) in absolute values, presented as individual data before (Pre) and after (Post) 11–12 weeks of strength training. Relative change presented as mean ± SD. *** = significant change from baseline (p < 0.001).

The training intervention resulted in a time effect (p < 0.001, Fig. 2A) and age effect (p < 0.05) in lean leg mass. Both young and old adults increased lean leg mass (1.1 ± 0.8 kg and 0.9 ± 0.5 kg, respectively, p < 0.001) and there were no differences between the groups in the relative change.

For changes in 1RM, the training intervention resulted in an interaction effect between time and age (p < 0.001, Fig. 2B), a time effect (p < 0.001) and an age effect (p < 0.001). Both groups increased leg press strength, and the young increased more than the old in absolute values (76.5 kg vs 50.5 kg, p < 0.01), whereas the relative change was not different between groups.

### 3.2 Training-induced changes in AATs and regulators of AATs

All factor and interaction effects from two-way ANOVA and unpaired t-tests for training-induced changes are listed in Supplementary Table S4.

LAT1 intensity in the membrane (M) and intracellular (IC) compartments by immunofluorescence microscopy (IF)

**Fig. 3:**
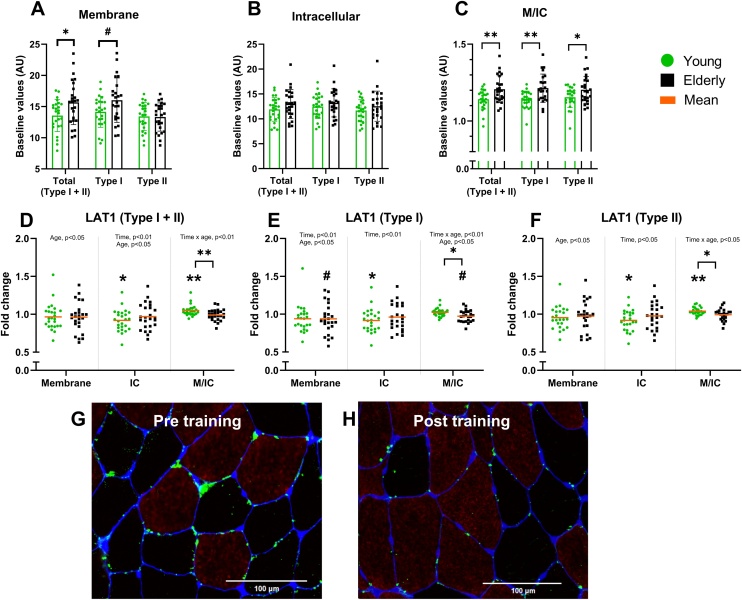
Immunofluorescent detection of LAT1 in young and old vastus lateralis muscle co-stained with myosin heavy chain 1 (MHC1) and WGA (DYS). Sections were stained with anti-LAT1 antibody (green) and then co-stained with dystrophin (blue) for the identification of the sarcolemma and MHC1 (red) for the identification of Type I. Baseline values are shown for (A) Membrane (M), (B) Intracellular (IC) and (C) M/IC-ratio, where significant differences between young and older adults are marked. Fold changes in mean intensity from before to after the training intervention are presented as mean ± SD for (D) Total LAT1, (E) Type I LAT1 and (F) Type II LAT1, where significant changes are marked within group and between young and older adults. ** = p < 0.01, * = p < 0.05 and # = p < 0.1.

Baseline values of LAT1 membrane intensity in all fibers were 15% higher (p < 0.05) and Type I fiber LAT1 tended to be higher (p = 0.07) in the older participants, compared to the young (Fig. 3A). Further, in old compared to young, the LAT1 M/IC-ratio was 7% and 4% higher in Type I (p < 0.001) and in Type II (p < 0.05), respectively (Fig. 3C). Sex specific explorative t-tests analyses revealed ∼20% higher baseline values of membrane LAT1 in Total (p < 0.05, Supplementary Fig. S2A), and Type I (p < 0.05) and a tendency to be 17% higher in Type II (p = 0.052) for the old, compared to young, men, whereas no differences were observed between young and old women. When exploring sex differences, we observed 17% lower membrane LAT1 intensity in Type I fibers (p < 0.05, Supplementary Fig. S2B), and tendencies for lower membrane LAT1 intensity in Type II fibers (p = 0.08), in old women, compared to old men at baseline.

The training intervention caused increases in the LAT1 M/IC ratio for the young and nonsignificant decreases for the old, which resulted in an interaction effect between time and age for all fibers (p < 0.01, Fig. 3D), Type I fibers (p < 0.01, Fig. 3E) and Type II fibers (p < 0.05, Fig. 3F). We also observed a time effect in LAT1, with decreased intensity, in the IC region in all fibers (p < 0.01, Fig. 3D), and in fiber-specific (Type I: p < 0.01, Type II: p < 0.05, Fig. 3E-F), as well as decreased intensity in the membrane region for Type I only (p < 0.01, Fig. 3E). An age effect was present in total LAT1 intensity in the membrane and the IC region (p < 0.05, Fig. 3D-F), as well as in membrane region only for Type I (p < 0.05, Fig. 3E) and Type II (p < 0.05, Fig. 3F), where the old showed higher intensity than the young. In the M/IC ratio, we observed an age effect in Type I fibers (p < 0.05, Fig. 3E), with higher intensity for the young than old.

In the IC region, the young decreased LAT1 intensity in all fibers (−9 ± 14%, p < 0.05, Fig. 3D) and in fiber-specific LAT1 intensity (Type I: −8 ± 16%; Type II: −8 ± 14%; p < 0.05, Fig. 3E-F). The old group tended to decrease LAT1 intensity in the membrane region of Type I fibers (−6 ± 20%, p = 0.09, Fig. 3E). In young, this led to a 5 ± 7% increase in the M/IC-ratio for LAT1 intensity for all fibers (p < 0.01, Fig. 3D) and a 4 ± 5% decrease in Type II fibers (p < 0.01, Fig. 3F), whereas the old tended to decrease the LAT1 M/IC-ratio in Type I fibers by 3 ± 8% (p = 0.07, Fig. 3E). After the training intervention, we observed 15–16 % higher total LAT1 intensity in both the membrane region and the IC region in the old compared to young (p < 0.05). When comparing the relative changes between fiber types in each group, we observed that LAT1 intensity in the old group decreased more in Type I fibers, compared to Type II, in the membrane region (−6 ± 20% vs −3 ± 20%, p < 0.01) and in the M/IC-ratio (−3 ± 8% vs −1 ± 8%, p < 0.01), whereas the LAT1 M/IC-ratio increased in Type II fibers, compared to Type I, for the young group (4 ± 5% vs 3 ± 6%, p < 0.05).

#### 3.2.1 AAT protein levels in the subcellular fractions

**Fig. 4:**
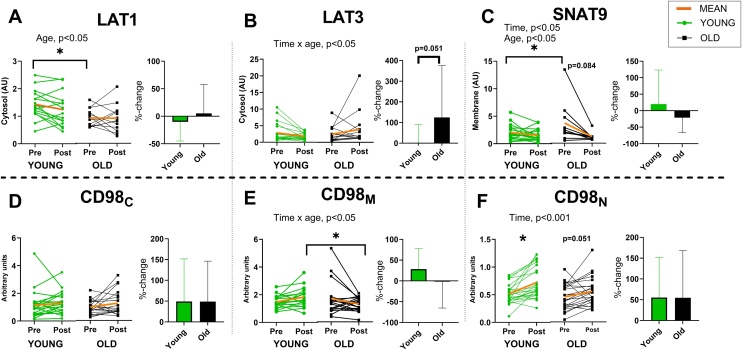
Protein levels of LAT1 in cytosol (A), LAT3 in cytosol (B), SNAT9 in membrane (C) and CD98 in cytosol (D), membrane (E) and nuclear (F) fraction before and after the training intervention for young and old adults. Presented as individual data in arbitrary units. Representative bands are shown in Supplementary Table S3. Relative changes (%) before and after the training intervention are presented as mean ± SD. * = significant change (p < 0.05).

Before training, we observed that the old had higher membrane SNAT9 and lower cytosolic LAT1 baseline levels, compared to young adults (p < 0.05, Fig. 4A and C). The sex specific analyses revealed ∼43% and ∼51% lower baseline levels of LAT1 in old women, compared to old men (p < 0.01, Supplementary Fig. S2C) and to young women (p < 0.05, Supplementary Fig. S2D) respectively. An interaction effect between time and age was found for training induced changes in AAT protein levels in membrane CD98 protein levels (CD98_M_), with a non-significant 28% increase in the young and no change in the old (p < 0.05, Fig. 4E), and in cytosolic LAT3 protein levels, with a non-significant 127% increase in the old and no change in the young (p < 0.05, Fig. 4B). We also observed a time effect for SNAT9 (p < 0.05) and nuclear CD98 protein levels (CD98_N_, p < 0.001), and an age effect for SNAT9 (p < 0.05) and LAT1 (p < 0.05).

During the training period, SNAT9 protein levels tended to decrease and were 21 ± 45% lower than at baseline after the training period in the old group (p = 0.084, Fig. 4C). For the young, the training intervention caused a 55 ± 97% increase in CD98_N_ (p < 0.001), and the old tended to increase CD98_N_ similarly by 55 ± 114% (p = 0.051, Fig. 4F). In the relative changes in LAT3 there was a tendency towards a difference between young and old (p = 0.084, Fig. 4B) as well as a tendency for older women to increase more than older men (266 ± 357% vs 30 ± 74%, p = 0.09). We also observed 41% higher levels of CD98_M_ in young compared to old adults (p < 0.05, Fig. 4E) after the training. The baseline differences between older women and men in LAT1 were abolished after the training, caused by a nonsignificant increase in LAT1 by 20 ± 48% for the old women and no change (−8 ± 56%) in old men, whereas it tended to remain different compared to young women (p = 0.06).

For training induced changes in regulators of AATs, we observed a time effect for nuclear eIF2α (eIF2α_N_, p < 0.001), cytosolic ATF4 (ATF4_C_, p < 0.05) and nuclear ATF4 (ATF4_N_, p < 0.01). For the young, the training intervention caused a 41 ± 66% increase in eIF2α_N_ (Supplementary Fig. S1, p < 0.05). The old group increased eIF2α_N_ by 65 ± 98% (p < 0.001) and ATF4_N_ by 191 ± 362% (Supplementary Fig. S1, p < 0.05) and tended to increase ATF4_C_ by 541 ± 1191% (p = 0.081) after the training period. After the training we also saw lower levels of eIF2α_N_ in the young compared to old (p < 0.05) and we observed a tendency to differ in the relative changes in membrane eIF2α (p = 0.056) between the groups.

## 4 Discussion

Our study aimed to investigate the long-term effects of strength training combined with protein supplementation on levels and location of AATs in young and older participants. To our knowledge, this is the first report on changes in protein levels for LAT3 and SNAT9 in relation to strength training and protein supplementation in human skeletal muscle, as well as the first comparison between young and older adults for LAT1 membrane intensity. We are further reporting on changes in fraction-specific levels of the investigated proteins, including cytosol, membrane, and nuclear fractions.

We hypothesized that older adults would have less AATs than young adults, which could be associated with a reduced anabolic response in older adults, and that these levels would recover with regular strength training. In contrast to our hypotheses, we observed that untrained, old adults had higher LAT1 intensity in the membrane region of the fibers, as well as higher protein levels of membrane SNAT9, compared to the young, and it was only the protein levels of cytosolic LAT1 that were lower in the old.

Previous studies comparing AATs in young and older adults, measured in homogenate, sarcoplasm or cytosol, observed no differences [14,36,37], or higher [13] basal levels of LAT1 in old compared to young adults. Higher AAT levels in older adults could indicate a compensatory mechanism to address insufficient amino acid delivery into the muscle fibers, which may be counteracted by sufficient protein intake. In support of this rationale is the observed reduction in LAT1 protein levels in older males when consuming 1.6, compared to 0.8, g·(kg body mass)^−1^ protein over a period of ten weeks [38]. The high general recommendation for increased protein intake in old adults, in order to mitigate sarcopenia, may therefore be sufficient to also eliminate differences in muscle AAT with aging [39]. The higher-than-expected AAT levels could therefore reflect an insufficient habitual protein intake, as nine of our older participants were reported to ingest less than 1.0 g·(kg body mass)^−1^ protein per day [31]. Low intracellular amino acid concentrations upregulate GAAC, which leads to selective expression of AATs together with a general mRNA translation inhibition [11]. Consequently, higher baseline protein levels of SNAT9 and membrane LAT1 intensity in the old compared to young adults could be a compensating mechanism to low initial amino acid availability in the muscle cell for the older group.

As expected, the training intervention induced increases in muscle mass and muscle strength for both young and old adults. These changes were accompanied by reduced LAT1 intensity in the intracellular region for the young, whereas the old trended to reduce SNAT9 membrane protein levels and LAT1 intensity in Type I fibers in the membrane region. LAT1 protein levels, measured with immunoblot, and counted LAT1 per fiber, measured with immunofluorescence, has previously been reported to increase for young adults following 12 weeks of strength training, with the suggestion that the increase is located internally in the cell [16]. A different study on young adults reported no change in LAT1 after eight weeks of strength training and increased protein intakes [15]. The protein intake and training intervention for the young in our study was similar to Roberson et al. (2020) and Abou Sawan et al. (2021), so discrepancies could be related to analytical differences or number of participants [15,16]. We show that cytosolic LAT1 protein levels are non-significantly reduced for the young, which agrees with our findings of reduced LAT1 intensity in the intracellular compartment. Roberson et al. (2020) showed that providing young adults with leucine or whey supplements during 12-weeks strength training resulted in a considerably lower increase in LAT1 when compared to the young that received placebo, making a connection between LAT1 levels and sufficient leucine intakes [16]. Abou Sawan et al. (2021) also showed that a intake of 1.2–1.5 g·(kg body mass)^−1^ during eight weeks of strength training did not change LAT1 protein levels for young [15]. Therefore, the observed reduction we observed in LAT1 intensity for the young in the intracellular region of the muscle fiber may be related in part to the increased protein intake during the intervention, even though the intake was also considerably high before the training (∼1.5 vs. 2.0 g·(kg body mass)^−1^) [31]. Previous reports of reduced LAT1 protein levels in older adults with increased protein intake suggests that less AATs are needed when sufficient protein is provided ^36^. The protein intake for our old participants was higher during the training in relation to the recommended protein intake (∼1.0 before vs 1.3 during, g· (kg body mass)^−1^) [32], but our results indicate that this was insufficient to cause a reduction in AATs. Considering that the intervention included both strength training and protein supplementation, the maintained LAT1 levels in older adults could be related to the addition of greater energy requirement with resistance training or a different adaptation in young and old adults to strength training. Further, the responses in AATs for young and old may come as a result of differential regulation with aging as is supported by our data.

During the training intervention, the differences between young and old adults in SNAT9 and cytosolic LAT1 disappeared, whereas the higher membrane LAT1 intensity in the old remained. The abolished difference between young and old adults in SNAT9 after training and protein supplementation could indicate an improved access to intracellular amino acids, such as leucine, in the old group, and thereby reducing the need for elevated AAT levels. Strength training may enhance the efficiency of AATs and/or boost AA availability in the muscle by reutilizing AA from protein breakdown more efficiently [40]. Combined with increased protein intake, this may account for the reduction in intracellular LAT1 and the modest non-significant decrease in cytosolic LAT1 for the young, as well as the absence of any change for the old. The age of the old participants may also be of importance, as age-related differences in AATs may not manifest until later stages than previously investigated (typically aged 60–75 years). Dramatic changes in muscle at certain ages are indicated by the increased prevalence of sarcopenia, which is the age-related loss in skeletal muscle mass and function, rising from 6 to 22% for the age group 60–70 years to 50% in those older than 80 years [41]. This could explain in part why we, in our study, observe an age difference in membrane LAT1 intensity, independent of training status. It is also plausible that membrane LAT1 content is related to muscle fiber size, given that measured intensity is area dependent. Older adults generally have smaller muscle fibers than younger adults, and women tend to have smaller muscle fibers compared to men [42,43]. Lower LAT1 protein levels have also been reported in women compared to men, regardless of training status [15]. Our sub-analysis revealed more LAT1 in old men, compared to young men or older women, whereas there were no differences between young women and men. These findings suggest that sex may play a role in AAT expression, warranting further investigation to confirm their biological relevance.

Most investigations of AAT protein levels—except for one acute study—have focused on whole-cell lysates, sarcoplasmic, or cytosolic fractions. However, changes in the membrane fraction are likely to be more functionally relevant. In the membrane fraction, we observed a tendency in the old for reduced SNAT9. SNAT9 activates mTORC1 pathway by transport of lysosomal leucine in a arginine-regulated manner and lower SNAT9 levels could indicate reduced potential for mTORC1 activation [27]. In line with this, the resting phosphorylation of p70S6K did decrease during the training intervention [32]. Considering that amino acid availability is high during the intervention, the mTORC1 activation may be less dependent on the AA transport by SNAT9, causing the observed reduction. However, our analyses do not distinguish between membranes in the cell, implying that our observations could be related to SNAT9 in other compartments of the cell such as the plasma membrane. Interestingly, the training intervention led to changes in nuclear protein levels of eIF2α and CD98 for both groups and in ATF4 for the old group only. The findings of strong nuclear bands for CD98 and eIF2α were surprising, and the function is currently unclear. However, increased nuclear ATF4 levels could indicate an increased activity of AAT transcription. Further, the older adults tended to increase more in cytosolic LAT3. Assessing multiple subcellular fractions may further clarify potential translocation dynamics of AATs and provide deeper insight into their regulatory roles in amino acid transport.

Our study has limitations in sample size calculations as this was not based on changes in AATs. We further acknowledge the exploratory nature of correlations and that we cannot directly determine the biological effect as we did not use activity assays and/or tracers.

## 5 Conclusion

In the untrained state, LAT1 protein was lower in the cytosol, whereas SNAT9 and LAT1 were higher in the membrane, in older adults compared to young, whereas no differences were observed for LAT3 and CD98. Strength training abolished the difference between young and older adults in SNAT9 membrane levels, but the membrane LAT1 content remained higher in older adults, and membrane CD98 became higher in the young after the training period. The observed sex differences in LAT1 at baseline between older women and men was further abolished after training. The regulation of AATs varies in how they are affected by aging and long-term exercise, which warrants future investigations of several AATs as well as to confirm the findings in this study.

## CRediT authorship contribution statement

**Elise Lander**: Data curation; Formal analysis; Investigation; Methodology; Validation; Visualization; Writing - original draft. **Håvard Hamarsland:** Writing - Review & Editing, Project administration; Methodology. **Matthew Lees**: Methodology; Validation, Writing - Review & Editing. **Daniel Moore**: Methodology, Writing - Review & Editing. **Truls Raastad:** Writing - Review & Editing, Supervision, Funding acquisition, Conceptualization.

## Declaration of Generative AI and AI-assisted technologies in the writing process

Not applicable.

## Funding

The main financial support was given by the 10.13039/501100005416Research Council of Norway (project number 225258/E40) and Tine SA, with support in part from a Natural Sciences and Engineering Research Council Discovery Grant (No. RGPIN-2023-05382) awarded to Daniel Moore.

## Data availability

Data will be made available on request.

## Declaration of competing interest

The authors report no declarations of interest.
