# Supplementary material for: Resistance training partially restores age-related differences in skeletal muscle amino acid transporters - secondary analysis from two randomized controlled trials
Source: J Nutr Health Aging. 2026 Feb 21;30(4):100808. doi: 10.1016/j.jnha.2026.100808 (PMC12945623; doi:10.1016/j.jnha.2026.100808)
Supplement: Supplementary file 1 [file mmc1.docx]

Supplementary materials

**Table 1.** Overview of antibodies used in immunohistochemistry and the concomitant protocols. NGS= normal goat serum. PBS= Phosphate buffered saline. N= number of participants in analyzes.

|  | **Staining protocol** | **LAT1** | **MHC1** | **Sarcolemma membrane** |
| --- | --- | --- | --- | --- |
| **Fixation and block** | **Fixation solution** | Acetone and ethanol in 3:1 solution (Fisher, Loughborough, UK) | | |
|  | **Fixation time** | 5 minutes | | |
|  | **Blocking buffer** | 5% NGS (GIBCO, 16210064) in PBS | | |
|  | **Blocking time** | 1 hour in room temperature | | |
| **Primary antibody (1ab)** | **Antibody** | Rabbit Recombinant Monoclonal SLC7A5/LAT1 antibody | Mouse monoclonal anti-Myosin Heavy Chain 1 (MHC1) antibody, isotype IgG2b | Wheat Germ Agglutinin-350 |
|  | **Producer** | Abcam | DSHB | Invitrogen |
|  | **Art.no.** | ab208776 | BA-F8-S | W11263 |
|  | **Lot no.** | GR3439651-2 |  | 16 |
|  | **Dilution of 1ab** | 1:100 | 1:100 | 1:20 |
|  | **1ab dilution buffer** | 5% NGS (GIBCO, 16210064) in PBS | | 1% BSA in PBS |
| **Secondary antibody (2ab)** | **Antibody** | Goat anti-rabbit IgG(H+L) Alexa®488 | Goat Anti-Mouse IgG H&L (Alexa Fluor® 594) | Alexa Fluor®350 Conjugated |
|  | **Producer** | Invitrogen | Abcam |  |
|  | **Art. no.** | A11008 | ab150116 |  |
|  | **Lot no.** | 2284595 | GR260592-3 |  |
|  | **Dilution of 2ab** | 1:200 | 1:200 | N/A |
|  | **2ab Dilution buffer** | 1% BSA in PBS | | N/A |
| **Image intensity** | **Light** | 61 | 35 | 13 |
|  | **Exposure** | 208ms | 130ms | 28ms |
|  | **Gain** | 3.2db | 3.0db | 2.5db |

**Table 2** Overview of antibodies used in western blot and the concomitant protocols. N indicates how many participants was included in each of the analyses. C= cytosol, M=Membrane, N=Nuclear. CST= Cell Signaling Technology. AFB= Animal free blocking. BSA= Bovine serum albumin. TBS-T= Tris-buffered saline with tween

|  | **Protein of interest:** | **eIF2α** | **CD98** | **ATF4** | **SLC38A9** | **LAT3** | **LAT1** |
| --- | --- | --- | --- | --- | --- | --- | --- |
| **Sample preparation** | **Loading volume** | 30 µl | | 20 µl | | | |
|  | **Loaded protein** | 9 µg | | 6 µg | | | |
|  | **End concentration (ug/ul)** | 0.3 µg/ul | | 0.3 µg/ul | 0.25-0.3 µg/ul | 0.3 µg/ul | |
|  | **Heating temperature** | 40°C | | 70°C | | | |
|  | **Heating time** | 30 minutes | | 10 minutes | | | |
| **Gel used in electrophoresis:** | | 4–20% precast polyacrylamide gel  (Bio-Rad, #4568093) | | 4–20% precast polyacrylamide gel  (Bio-Rad, #4568095) | | | |
| **Blott and block** | **Blotting program** | Low molecular weight | | Mixed molecular weight | | | |
|  | **Blotting time** | 5 minutes | | 7 minutes | | | |
|  | **Blocking buffer** | 5% milk in TBS-T | | AFB | 3% BSA in TBS-T | | |
|  | **Blocking time** | 2 hours | | | | | |
| **Primary antibody** | **Producer** | CST | Sigma-Aldrich | Abcam | Abcam | Sigma-Aldrich | Abcam |
|  | **Art.no.** | 5324 | SAB1400262 | ab1371 | ab130398 | SAB4503399 | ab99419 |
|  | **Dilution of 1ab** | 1:1000 | | 1: 2000 | 1: 250 | 1: 500 | 1:1000 |
|  | **1ab dilution buffer** | 1% milk in TBS-T | | AFB | 5% BSA in TBS-T | 1% BSA in TBS-T | 5% BSA |
| **Secondary antibody** | **Producer** | Thermo Fisher | CST | Abcam | CST | CST | Abcam |
|  | **Art. no.** | 31460 | 7074S | ab6741 | 7074S | 7074S | ab6741 |
|  | **Dilution of 2ab** | 1: 30 000 | 1: 1000 | 1:8000 | 1: 1000 | 1: 1000 | 1: 5000 |
|  | **2ab Dilution buffer** | 1% milk in TBS-T | | AFB | 1% BSA in TBS-T | 1% BSA in TBS-T | 1% BSA in TBS-T |
| **MW** | **Estimated molecular weight** | 38 | 80 | 37 | 64 | 61 | 55 |
|  | **Observed molecular weight** | 35 | 80 | 55 | 64 | 64 | 55 |

**Table 3** Representative bands of proteins analyzed with Western Blot. Baseline sample to the left and trained sample to the right for the groups separately. MW=Molecular weight.

| Protein | Fraction | Young cohort | | | | Old cohort | | | |
| --- | --- | --- | --- | --- | --- | --- | --- | --- | --- |
|  |  | Baseline | UnT.ex | Trained | T.ex | Baseline | UnT.ex | Trained | T.ex |
| SNAT9 | Membrane | 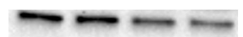 | | | | 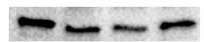 | | | |
| CD98 | Cytosol | 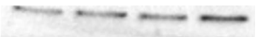 | | | | 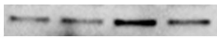 | | | |
|  | Membrane | 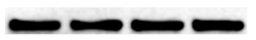 | | | | 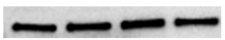 | | | |
|  | Nucleus | 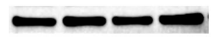 | | | | 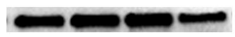 | | | |
| eIF2α | Cytosol | 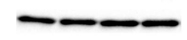 | | | | 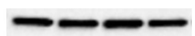 | | | |
|  | Membrane | 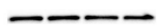 | | | | 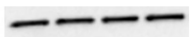 | | | |
|  | Nucleus | 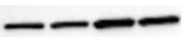 | | | | 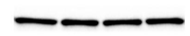 | | | |
| ATF4 | Cytosol | 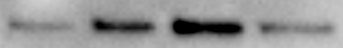 | | | | 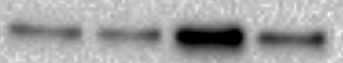 | | | |
|  | Nucleus | 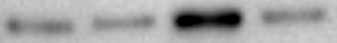 | | | | 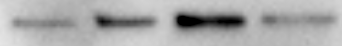 | | | |
| LAT3 | Cytosol | 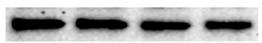 | | | | 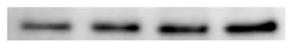 | | | |


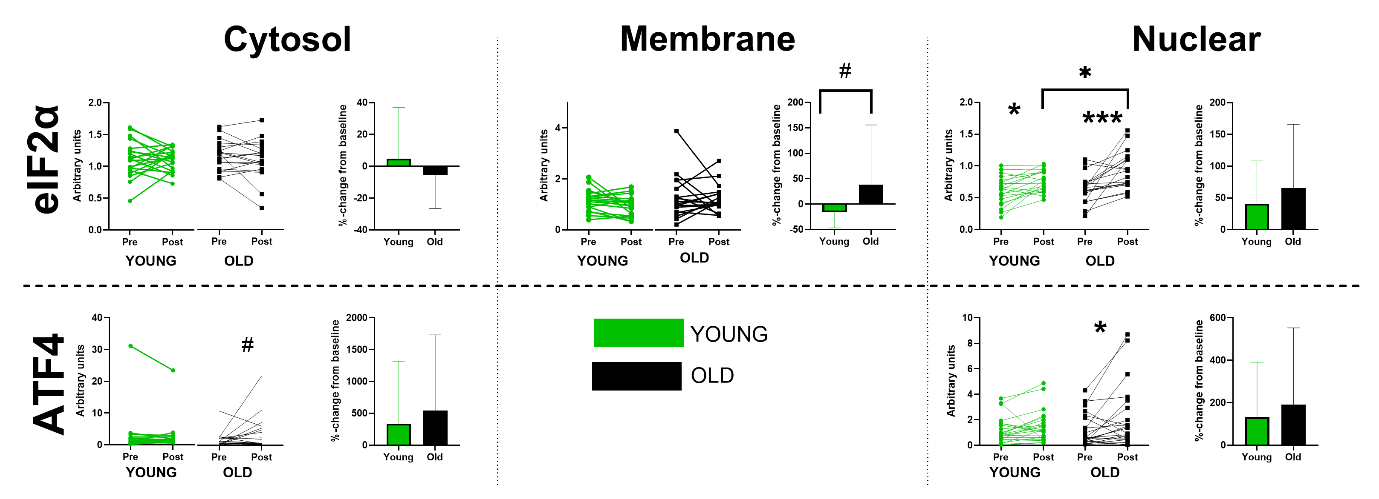
**Figure 1** Protein levels of eIF2α in cytosol, membrane and nuclear fraction and ATF4 in cytosol and nuclear fraction before and after the training intervention for young and elderly. Presented as individual data in arbitrary units. Relative changes (%) before and after the training intervention are presented as mean ± SD. *=significant change (p<0.05). ***=p<0.001. #=tendency to change (p<0.1)

**Table 4** P-values from statistical tests of western blot and immunohistochemistry (IHC) data for training-induced changes, using two-way ANOVA, except for Student’s t-test for unpaired samples when investigating the relative change (%) between young and elderly. Significant p-values are marked in bold. IC= intracellular. M= membrane.

|  |  |  |  | Training intervention | | | | | |
| --- | --- | --- | --- | --- | --- | --- | --- | --- | --- |
|  |  |  |  | N | | Arbitrary units | | | %-change |
|  | Protein | Fraction | Logtransferred | Young | Old | Time x Age | Time | Age | Young VS Old |
| Western Blot | CD98 | Cytosol | Yes | 22 | 20 | 0.837 | 0.055 | 0.852 | 0.995 |
|  |  | Membrane | Yes | 18 | 18 | **0.033** | 0.983 | 0.166 | 0.121 |
|  |  | Nucleus | No | 24 | 23 | 0.373 | **<.001** | 0.494 | 0.982 |
|  | eIF2α | Cytosol | No | 20 | 19 | 0.504 | 0.341 | 0.839 | 0.239 |
|  |  | Membrane | Yes | 19 | 18 | 0.097 | 0.352 | 0.364 | 0.056 |
|  |  | Nucleus | No | 20 | 19 | 0.105 | **<.001** | 0.105 | 0.378 |
|  | LAT3 | Cytosol | Yes | 20 | 15 | **0.028** | 0.573 | 0.505 | 0.051 |
|  | ATF4 | Cytosol | Yes | 18 | 19 | 0.629 | **0.018** | 0.458 | 0.554 |
|  |  | Nucleus | Yes | 23 | 23 | 0.779 | **0.003** | 0.931 | 0.565 |
|  | SNAT9 | Membrane | Yes | 22 | 17 | 0.401 | **0.025** | **0.019** | 0.130 |
|  | LAT1 | Cytosol | No | 16 | 13 | 0.261 | 0.229 | **0.019** | 0.351 |
| IHC | Total LAT1 | Membrane | No | 25 | 25 | 0.871 | 0.060 | **0.011** | 0.963 |
|  |  | Intracellular | No | 25 | 25 | 0.342 | **0.010** | **0.041** | 0.270 |
|  |  | M/IC |  | 25 | 25 | **0.006** | 0.094 | 0.102 | **0.006** |
|  | Type 1 LAT1 | Membrane | No | 24 | 24 | 0.861 | **0.009** | **0.024** | 0.928 |
|  |  | Intracellular | No | 24 | 24 | 0.374 | **0.007** | 0.125 | 0.341 |
|  |  | M/IC |  | 24 | 24 | **0.009** | 0.705 | **0.027** | **0.011** |
|  | Type 2 LAT1 | Membrane | No | 24 | 24 | 0.945 | 0.071 | **0.034** | 0.697 |
|  |  | Intracellular | No | 24 | 24 | 0.303 | **0.016** | 0.081 | 0.191 |
|  |  | M/IC |  | 24 | 24 | **0.022** | 0.152 | 0.314 | **0.022** |


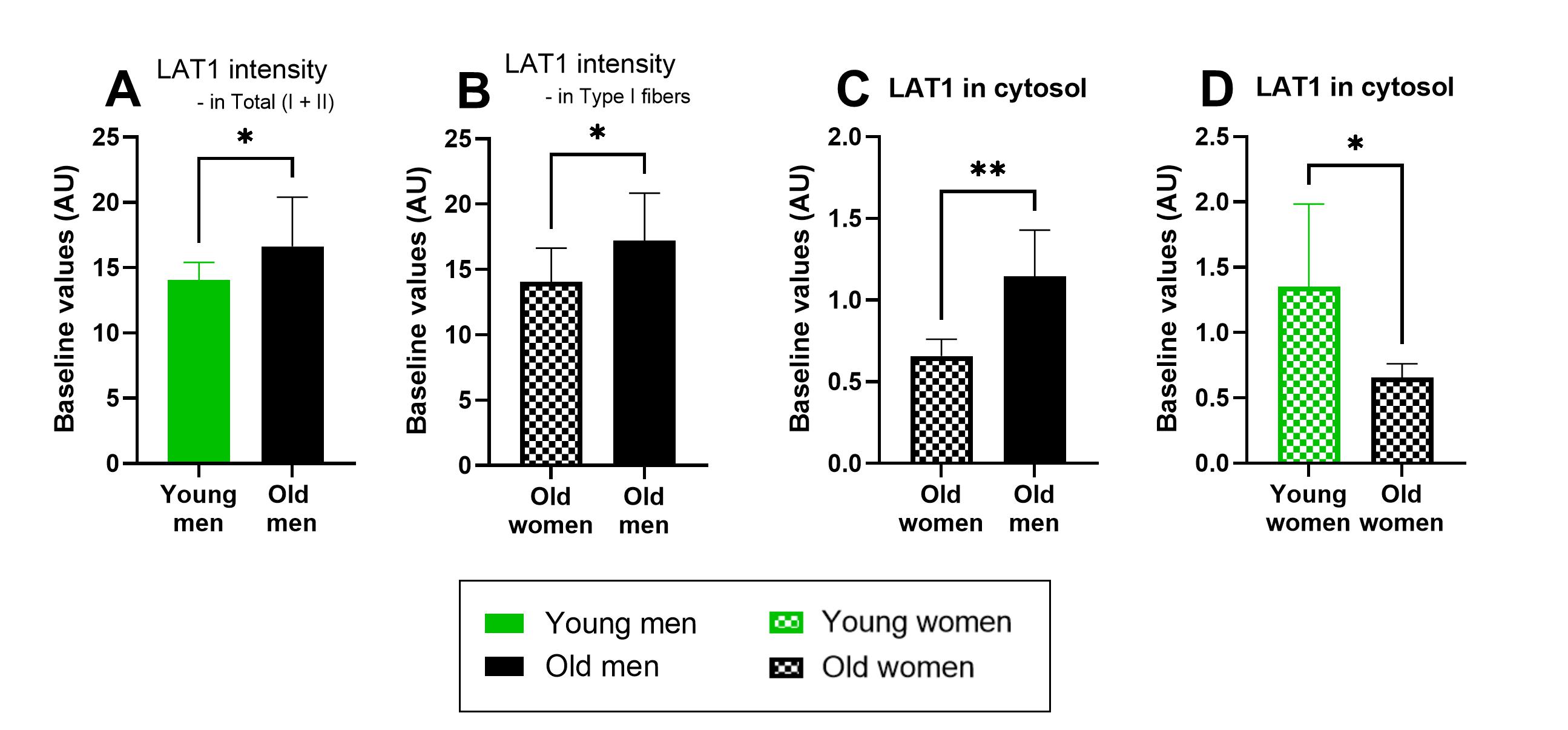


**Figure 2** Comparisons of baseline values in LAT1 intensity measured with immunohistochemistry between young and old men (A) and old women and men (B) and in cytosolic LAT1 protein levels measure with western blot between old women and men (C) and young and old women (D). Presented in arbitrary units as mean ± SD. *=significant change (p<0.05). **=p<0.01.
